# Genome Sequences of 15 Klebsiella sp. Isolates from Sugarcane Fields in Colombia’s Cauca Valley

**DOI:** 10.1128/genomeA.00104-18

**Published:** 2018-03-22

**Authors:** Luz K. Medina-Cordoba, Aroon T. Chande, Lavanya Rishishwar, Leonard W. Mayer, Leonardo Mariño-Ramírez, Lina C. Valderrama-Aguirre, Augusto Valderrama-Aguirre, Joel E. Kostka, I. King Jordan

**Affiliations:** a School of Biological Sciences, Georgia Institute of Technology, Atlanta, Georgia, USA; b PanAmerican Bioinformatics Institute, Cali, Valle del Cauca, Colombia; c Applied Bioinformatics Laboratory, Atlanta, Georgia, USA; d Laboratory of Microorganismal Production (Bioinoculums), Department of Field Research in Sugarcane, INCAUCA S.A.S., Cali, Valle del Cauca, Colombia; e Biomedical Research Institute, Universidad Libre, Cali, Valle del Cauca, Colombia; f National Center for Biotechnology Information, National Library of Medicine, National Institutes of Health, Bethesda, Maryland, USA; g School of Natural Resources and Environmental Engineering, Ph.D. Program in Sanitary and Environmental Engineering, Universidad del Valle, Cali, Valle del Cauca, Colombia

## Abstract

Members of the Klebsiella genus promote plant growth. We report here draft whole-genome sequences for 15 Klebsiella sp. isolates from sugarcane fields in the Cauca Valley of Colombia. The genomes of these isolates were characterized as part of a broader effort to evaluate their utility as endemic plant growth-promoting biofertilizers.

## GENOME ANNOUNCEMENT

The genus Klebsiella belongs to the family Enterobacteriaceae and includes nonmotile rod-shaped Gram-negative bacteria with polysaccharide capsules. Members of the Klebsiella genus are exceptionally widespread in nature; Klebsiella spp. inhabit both water and soil environments, and they are associated with numerous plant and animal species (1). Klebsiella spp. are known to promote plant growth by colonizing plant tissues (roots) and providing essential nutrients to their plant hosts (2). For example, Klebsiella spp. encode the biochemical capacity to fix nitrogen, i.e., to convert molecular nitrogen to organic nitrogen in the form of ammonium (3). Plant growth promotion can also be facilitated via a number of other mechanisms, including phosphate solubilization, the production of phytohormones, an increase in nutritional uptake, and control of environmental stress (4, 5). The aim of this project was to use the analysis of Klebsiella sp. isolate genome sequences to evaluate their potential as biofertilizers. Given the fact that some Klebsiella spp. are known (opportunistic) pathogens, genome sequence analysis can also be used to mitigate the potential risk they pose to human populations if included as part of a bioinoculum.

The 15 Klebsiella sp. isolates characterized here were isolated from INCAUCA sugarcane fields, either from plants’ root zones or directly from plant tissue. All isolates were grown overnight on LB medium (Difco) at 37°C. Genomic DNA was isolated using the E.Z.N.A. bacterial DNA kit (Omega Bio-tek), and paired-end fragment libraries were constructed using the Nextera XT DNA library preparation kit (Illumina), with a fragment length of 1,000 bp. Libraries were sequenced on an Illumina MiSeq platform using V3 chemistry, yielding approximately 400,000 paired-end 300-bp sequence reads per sample. Sequence read quality control was performed using the program FastQC version 0.11.5 (6). Adapter/primer sequences and low-quality bases and reads (Q < 20) were removed using Trimmomatic version 0.35 (7).

The 15 Klebsiella sp. isolate genomes were assembled using the *de novo* assembler SPAdes version 3.6 (8). The summary statistics for the resulting assemblies indicate the completeness of the work. The genome coverages range from 50× to 88×, with an average of 64× coverage, which is more than sufficient to produce reliable assemblies. Accordingly, the genome assembly metrics are robust; *N*_50_ values range from 65,329 bp to 614,324 bp, with an average *N*_50_ of 290,406 bp, and *L*_50_ values range from 3 to 29, with an average value of 8.9. Finally, the genome size and GC content values inferred from the assemblies are consistent with what is expected for Klebsiella species. Assembled genome sizes range from 5.46 Mb to 6.09 Mb, with an average size of 5.64 Mb, and the GC content values range from 56.7% to 57.5%, with an average GC content of 57.1%.

Isolate genome sequences were annotated using the Rapid Annotations using Subsystems Technology (RAST) Web server (9–11). Functional predictions will be used to prioritize strains that are simultaneously enriched for nitrogen fixing and other plant growth-promoting genes while containing minimal antibiotic resistance genes and virulence factors.

### Accession number(s).

This whole-genome shotgun project has been deposited at DDBJ/ENA/GenBank under the accession numbers shown in Table 1.

**Table 1:** Strains obtained from Saccharum hybrid cultivars in 2014 from Colombia and sequenced in this study

Strain	GenBank accession no.
A-Nf5	PIOG00000000
B-Nf7	PIOH00000000
C-Nf10	PIOJ00000000
C1-16S-Nf17	PIOI00000000
D-Nf1	PIOK00000000
E-Nf3	PIOL00000000
F-Nf9	PIOM00000000
G-Nf4	PION00000000
G2-16S-Nf13	PIOO00000000
H-Nf2	PIBL00000000
I-Nf8	PIBM00000000
J-Nf11	PJDI00000000
K-Nf6	PIBN00000000
T11	PJDJ00000000
X1-16S-Nf21	PJDK00000000
